# Comparison of Cup Position and Perioperative Characteristics in Total Hip Arthroplasty Following Three Types of Pelvic Osteotomy

**DOI:** 10.3390/medicina61081407

**Published:** 2025-08-02

**Authors:** Ryuichi Kanabuchi, Yu Mori, Kazuyoshi Baba, Hidetatsu Tanaka, Hiroaki Kurishima, Yasuaki Kuriyama, Hideki Fukuchi, Hiroki Kawamata, Toshimi Aizawa

**Affiliations:** Department of Orthopaedic Surgery, Tohoku University Graduate School of Medicine, Sendai 980-8574, Japan; ryuichi.kanabuchi.b8@tohoku.ac.jp (R.K.); kazuyoshi.baba.e3@tohoku.ac.jp (K.B.); hidetatsu.tanaka.c7@tohoku.ac.jp (H.T.); marronile@gmail.com (H.K.); yasuaki.kuriyama.b5@tohoku.ac.jp (Y.K.); hf111503@yahoo.co.jp (H.F.); hiroki.kawamata.e4@tohoku.ac.jp (H.K.); toshimi.aizawa.a5@tohoku.ac.jp (T.A.)

**Keywords:** developmental dysplasia of the hip, total hip arthroplasty, periacetabular osteotomy, Chiari osteotomy, shelf procedure, cup height, conversion surgery

## Abstract

*Background and Objectives*: Total hip arthroplasty (THA) following pelvic osteotomy for developmental dysplasia of the hip (DDH) is technically challenging due to altered acetabular morphology. This study aimed to compare radiographic cup position and perioperative characteristics of THA after three common pelvic osteotomies—periacetabular osteotomy (PAO), shelf procedure, and Chiari osteotomy—with primary THA in Crowe type I DDH. *Methods:* A retrospective review identified 25 hips that underwent conversion THA after pelvic osteotomy (PAO = 12, shelf = 8, Chiari = 5) and 25 primary THAs without prior osteotomy. One-to-one matching was performed based on sex (exact match), age (within 5 years), and BMI (within 2 kg/m^2^) without the use of propensity scores. Cup inclination, radiographic anteversion, center-edge (CE) angle, and cup height were measured on standardized anteroposterior radiographs (ICC = 0.91). Operative time, estimated blood loss, and use of bulk bone grafts or reinforcement rings were reviewed. One-way ANOVA with Dunnett’s post hoc test and chi-square test were used for statistical comparison. *Results:* Cup inclination, anteversion, and CE angle did not differ significantly among groups. Cup height was significantly greater in the PAO group than in controls (29.0 mm vs. 21.8 mm; *p* = 0.0075), indicating a more proximal hip center. The Chiari and shelf groups showed upward trends, though not significant. Mean operative time tended to be longer after PAO (123 min vs. 93 min; *p* = 0.078). Bulk bone grafts and reinforcement rings were more frequently required in the PAO group (17%; *p* = 0.036 vs. control), and occasionally in Chiari cases, but not in shelf or control groups. *Conclusions:* THA after PAO is associated with higher cup placement and greater need for reconstructive devices, indicating increased technical complexity. In contrast, shelf and Chiari conversions more closely resemble primary THA. Preoperative planning should consider hip center translation and bone-stock restoration in post-osteotomy THA.

## 1. Introduction

Developmental dysplasia of the hip (DDH) encompasses a range of hip joint abnormalities, from subtle acetabular underdevelopment to complete dislocation of the femoral head [1,2,3]. DDH is a multifactorial condition influenced by genetic, mechanical, and environmental factors [4,5,6,7,8,9,10,11]. Genetic predisposition plays a significant role, with a positive family history conferring a 12-fold increased risk [12]. Specific gene mutations, such as those affecting growth differentiation factor 5 (*GDF5)*, asporin, and C-X3-C motif chemokine receptor 1 (*CX3CR1)*, have also been associated with DDH development [1,5,6,7,8]. A genome-wide association study has also reported a link between ferroptosis and DDH [9]. Mechanical factors are equally critical [3,12]. Intrauterine positioning—particularly breech presentation—can restrict fetal mobility and lead to abnormal hip development. Breech presentation is present in up to 30–50% of DDH cases, with affected infants facing a risk as high as 25% [12]. Firstborn infants and those with high birth weight are at increased risk, likely due to limited intrauterine space. Oligohydramnios, characterized by reduced amniotic fluid, further contributes to constrained fetal movement and an increased risk of DDH [12]. Postnatally, cultural practices such as swaddling infants with hips in an extended and adducted position have been recognized as modifiable risk factors contributing to hip instability [3,12].

**Figure 1:**
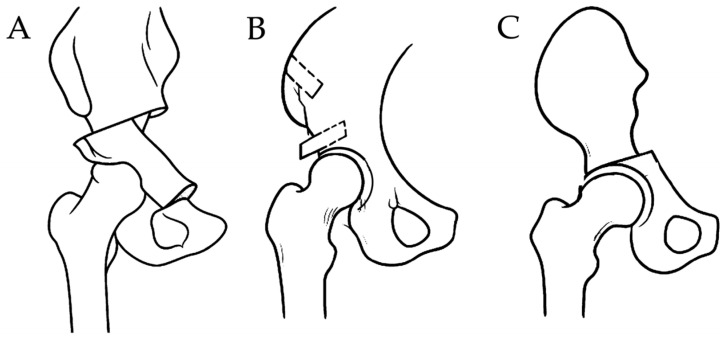
Schematic illustrations of three pelvic osteotomy types ((**A**) Periacetabular osteotomy, (**B**) Shelf procedure, (**C**) Chiari osteotomy), showing characteristic changes in acetabular morphology. These diagrams are provided to clarify the expected anatomical alterations that may affect subsequent total hip arthroplasty.

DDH often leads to secondary osteoarthritis due to structural instability of the acetabulum [13]. To prevent disease progression, various types of pelvic osteotomies such as periacetabular osteotomy (PAO; also known as the Bernese or Ganz osteotomy) [14,15], the shelf procedure [16,17], and Chiari osteotomy [18,19] have been developed to improve joint congruency and mechanical loading. A schematic illustration of the pelvic osteotomy procedures is presented in Figure 1. The severity of dysplasia, often classified using the Crowe system, plays a critical role in surgical decision-making. For example, Crowe type I or II hips with preserved femoral head coverage may be suitable for joint-preserving osteotomies such as the shelf procedure or PAO, while more advanced deformities (e.g., type III or IV) may necessitate alternative surgical strategies or direct conversion to total hip arthroplasty (THA) [20]. However, when osteoarthritic changes still occur, THA is indicated. THA following pelvic osteotomy presents unique technical challenges due to altered bony morphology, scarring, and potential distortion of anatomical landmarks.

Prior pelvic osteotomies significantly alter the native acetabular anatomy, complicating the identification of anatomical landmarks and the achievement of optimal component positioning during THA. Postoperative scarring and soft tissue change further obscure dissection planes, contributing to increased operative time and intraoperative blood loss. The distortion or absence of conventional reference points heightens the risk of component malpositioning, particularly with respect to cup anteversion and abduction angles. Moreover, compromised bone stock or residual dysplasia may impede secure acetabular component fixation. These anatomical alterations frequently result in a more proximal and lateral placement of the hip center, potentially compromising joint biomechanics and the long-term durability of the implant [21,22]. In particular, lateral displacement may reduce the mechanical advantage of the abductor muscles and increase joint reaction forces, which can lead to compromised gait mechanics, accelerated polyethylene wear, or postoperative instability [23,24].

THA after pelvic osteotomy presents several intraoperative and postoperative challenges. Intraoperatively, procedures are characterized by prolonged operative time and increased blood loss. Accurate component positioning is more difficult, with increased variability in cup orientation and a tendency toward reduced anteversion and proximal or lateral displacement of the hip center. Functional outcomes, including scores such as the Harris Hip Score, are modestly reduced compared to primary THA, though differences may not always reach clinical significance. Dislocation remains the most common complication, while overall complication and revision rates are inconsistently higher, with some studies indicating increased rates of aseptic loosening. Advanced imaging modalities, particularly computed tomography, and meticulous preoperative planning are essential to accommodate altered anatomy and optimize surgical outcomes.

This study aims to investigate and compare the intraoperative characteristics and radiographic outcomes of THA following three distinct types of pelvic osteotomy—PAO, the shelf procedure, and Chiari osteotomy—in patients with a history of DDH. Specifically, it seeks to evaluate differences in operative time, blood loss, cup positioning parameters (anteversion, inclination, and hip center position), and short-term postoperative outcomes among these groups. We hypothesized that different types of prior pelvic osteotomy would influence the complexity and radiographic outcomes of subsequent THA. Based on prior anatomical studies, we anticipated that THA following PAO might be associated with higher cup placement and increased use of reconstructive devices due to residual segmental defects and altered acetabular orientation, whereas Chiari and shelf procedures would lead to more modest deviations.

## 2. Materials and Methods

### 2.1. Patients

This retrospective study adhered to the ethical principles stated in the Declaration of Helsinki and was approved by the ethical review boards of Tohoku University (approval number: No. 2021-1-1059). Informed consent was taken from all patients.

This retrospective study included patients who underwent THA at our institution. The post-osteotomy THA group consisted of patients who received THA following a previous pelvic osteotomy between April 2003 and March 2020. The primary THA group, serving as the control group, included patients who underwent primary THA for Crowe type I DDH, without any history of pelvic osteotomy, who underwent primary THA between April 2014 and March 2021. Matching was performed in a 1:1 ratio without using propensity scores. Patients were matched based on sex (exact match), age (within ±5 years), and body mass index (within ±2 kg/m^2^) to ensure comparability between groups. Exclusion criteria were a follow-up period of less than one year and a history of revision THA. To eliminate the influence of cement use on operative time, THA cases involving cemented fixation were excluded. This decision was made to reduce procedural variability and ensure consistency across groups. Although no formal preliminary analysis was conducted, institutional experience indicated that cementation may prolong operative time and introduce technical differences that could confound intergroup comparisons. After applying these criteria, 50 patients were included in the final analysis, with 25 patients in each group. In addition to the history of pelvic osteotomy, patient characteristics, including sex, age at the time of THA, and body mass index, were collected and analyzed to ensure baseline comparability among groups.

### 2.2. Radiographic Evaluation

This study specifically focused on the radiographic assessment of acetabular cup positioning. The accuracy of femoral stem alignment and positioning was not evaluated. Cup positioning was assessed using standardized anteroposterior (AP) pelvic radiographs taken in the supine position. The following radiographic parameters were measured:

I. Cup inclination angle: Defined as the angle between the inter-teardrop line and the opening face of the acetabular component. This reflects the abduction angle of the cup and was measured directly on the AP radiograph.
II. Center-edge (CE) angle: Measured between a vertical line drawn from the center of the femoral head and a line connecting the femoral head center to the lateral edge of the acetabular cup. This parameter was used to evaluate the degree of lateral coverage of the implant.
III. Cup height: Defined as the vertical distance between the center of the femoral head (or the prosthetic head) and the inter-teardrop line. In all cases, the inter-teardrop line was identifiable on standardized supine AP pelvic radiographs. In cases with mild distortion, contralateral bony landmarks and pelvic symmetry were used to confirm the accuracy of reference points.
IV. Radiographic anteversion: Estimated using Lewinnek’s method [25], calculated as the inverse sine of the ratio of the short axis (*D1*) to the long axis (*D2*) of the elliptical projection of the cup on the AP radiograph:

$$\mathrm{Anteversion}=\sin^{-1}(\frac{D1}{D2})$$

where *D1* is the minor axis (short diameter) and *D2* is the major axis (long diameter) of the elliptical outline of the acetabular component.

**Figure 2:**
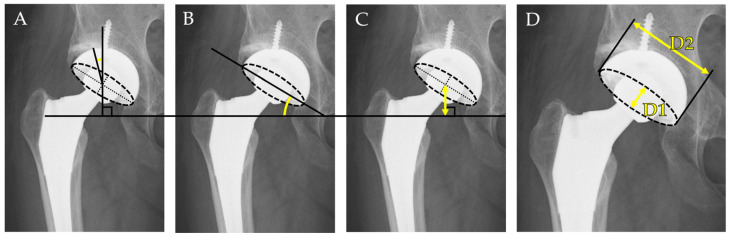
Radiographic measurement of acetabular cup positioning. (**A**) Center-edge (CE) angle: the angle formed between a vertical line perpendicular to the inter-teardrop line and a line connecting the center of the femoral head to the original lateral edge of the native acetabulum. (**B**) Inclination angle: the angle between the inter-teardrop line and the opening face of the acetabular component. (**C**) Cup height: the vertical distance from the femoral head center to the inter-teardrop line. (**D**) Radiographic anteversion: calculated using Lewinnek’s method as sin^−1^ ($\frac{D1}{D2}$), where *D1* and *D2* represent the short and long axes of the elliptical projection of the cup on anteroposterior radiographs.

The measurement methodology, including the evaluation of cup positioning, is outlined in Figure 2. All measurements were performed independently by two observers, and the mean values were used for analysis to minimize interobserver variability.

### 2.3. Surgical Characteristics

The following surgical parameters were evaluated to assess intraoperative complexity and technical demands of THA:

I. Operative time: Defined as the duration from skin incision to wound closure, recorded in minutes, as documented in the operative records.
II. Estimated blood loss: Determined based on intraoperative suction volume and the weight of surgical sponges, as recorded in the anesthesia chart. This parameter was used to assess intraoperative bleeding associated with anatomical complexity or surgical difficulty.
III. Use of bulk bone grafting or reinforcement devices: These reconstructive measures were employed when acetabular bone stock was insufficient for stable component fixation due to prior osteotomy, dysplasia, or bone defects. Bulk bone grafts consisted of either morselized or structural autografts harvested from the resected femoral head, or in some cases, allografts. Reinforcement devices included acetabular support rings (e.g., KT plates) and cages, selected intraoperatively according to the extent of bone deficiency.

These parameters were extracted retrospectively from operative notes and anesthesia records and compared between groups to evaluate differences in surgical difficulty and reconstructive strategy.

### 2.4. Statistical Analysis

All continuous variables are presented as mean ± standard deviation. Inter-group comparisons of continuous variables were performed using one-way analysis of variance. When a significant overall effect was observed, Dunnett’s post hoc test was used to compare each group with the control group. To assess interobserver reliability of the radiographic measurements, intraclass correlation coefficients (ICCs) were calculated. An overall ICC was obtained across all radiographic parameters (cup height, inclination angle, and CE angle). All measurements were independently performed by two observers, and high ICC values confirmed the reproducibility and consistency of the radiographic assessments. For categorical variables, such as sex, comparisons were performed using the chi-square (χ^2^) test or Fisher’s exact test. All statistical analyses were conducted using JMP Pro software, version 18 (SAS Institute Inc., Cary, NC, USA). A two-tailed *p* value of less than 0.05 was considered statistically significant.

## 3. Results

### 3.1. Patient Background

A total of 50 patients were included in the study and divided into four groups: 12 patients in the PAO group, 5 in the Chiari group, 8 in the shelf group, and 25 in the control group (primary THA without prior pelvic osteotomy). There were no statistically significant differences among the four groups in terms of sex distribution, body mass index, or age at the time of THA. These results indicate that the baseline demographics were comparable across all groups. Detailed data are presented in Table 1.

Data are presented as mean ± standard deviation. *p*-values represent post hoc comparisons between each osteotomy group (PAO, shelf, Chiari) and the control group using Dunnett’s test following one-way ANOVA (for age and BMI) or the chi-square test (for sex), with *p*-values < 0.05 considered statistically significant. BMI: body mass index; THA: total hip arthroplasty.

### 3.2. Radiographic Measurements

**Figure 3:**
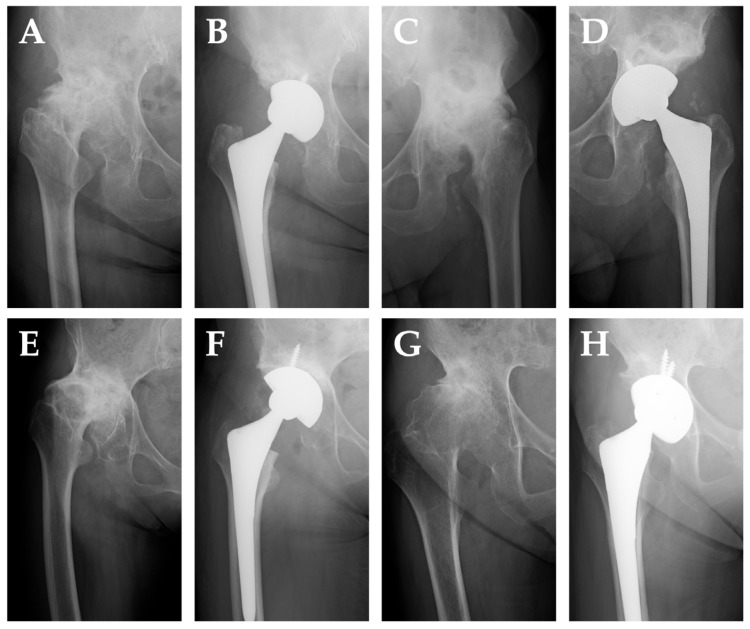
Representative anteroposterior radiographs of total hip arthroplasty (THA) cases. (**A**,**B**) A 62-year-old woman who underwent THA 17 years after periacetabular osteotomy (PAO). (**C**,**D**) A 66-year-old man who underwent THA 32 years after shelf procedure. (**E**,**F**) A 67-year-old woman who underwent THA 25 years after Chiari osteotomy. (**G**,**H**) A 68-year-old woman who underwent primary THA without a history of pelvic osteotomy.

Representative preoperative and postoperative radiographs of THA cases from the PAO, shelf, Chiari, and control groups are shown in Figure 3.

The results of radiographic cup positioning are summarized in Table 2. There were no statistically significant differences among the groups in cup inclination angle (PAO: 45.2°, shelf: 47.2°, Chiari: 39.2°, control: 43.7°) or radiographic anteversion (PAO: 13.9°, shelf: 14.4°, Chiari: 9.4°, control: 14.7°). Similarly, the center-edge (CE) angle did not differ significantly among the groups (PAO: 24.4°, shelf: 28.2°, Chiari: 18.0°, control: 18.1°). However, the vertical cup height from the inter-teardrop line was significantly greater in the PAO group (29.0 ± 6.7 mm) than in the control group (21.8 ± 4.9 mm; *p* = 0.0075), indicating a more proximal placement of the hip center in patients with prior periacetabular osteotomy. Although the Chiari and shelf groups also showed higher cup positions (27.8 mm and 27.2 mm, respectively), the differences did not reach statistical significance. To confirm measurement reproducibility, all radiographic parameters were independently assessed by two observers. The interobserver agreement was evaluated using the ICC, which yielded a value of 0.91, indicating excellent reliability.

Data are presented as mean ± standard deviation. *p*-values represent post hoc comparisons between each osteotomy group (PAO, shelf, Chiari) and the control group using Dunnett’s test following one-way ANOVA, with *p*-values < 0.05 considered statistically significant.

### 3.3. Surgical Parameters

Surgical outcomes are summarized in Table 3. The operative time tended to be longer in the PAO group (123.0 min) compared to the control group (93.1 min), although this difference did not reach statistical significance (*p* = 0.078). The shelf and Chiari groups had operative times of 107.2 and 97.2 min, respectively, which were comparable to the control group. There were no significant differences in estimated blood loss among the groups (PAO: 499.0 mL; shelf: 549.2 mL; Chiari: 329.1 mL; control: 466.0 mL). The use of bulk bone grafting was significantly more frequent in the PAO group (2/12 cases, *p* = 0.036 vs. control), while it was observed in only one case in the Chiari group and none in the shelf or control groups. Similarly, support ring usage was significantly higher in the PAO group (2/12 cases, *p* = 0.036 vs. control), whereas no support rings were used in the other three groups. These findings suggest that THA following PAO may require additional reconstructive procedures, such as bulk bone grafting and support ring application, more frequently than other osteotomy types or primary THA. No cases of postoperative hip dislocation were identified in any group during the available follow-up period. Other major complications (such as periprosthetic infection or aseptic loosening) were also not observed. Due to the small sample size, formal statistical comparison of complication rates was not performed.

Data are presented as mean ± standard deviation. *p*-values represent post hoc comparisons between each osteotomy group (PAO, shelf, Chiari) and the control group using Dunnett’s test following one-way ANOVA (for operative time and blood loss) or Fisher’s exact test (for categorical variables such as use of bulk bone grafts and support rings), with *p*-values < 0.05 considered statistically significant.

## 4. Discussion

This study compared radiographic cup position and intra-operative parameters of total hip arthroplasty performed after three common pelvic osteotomies for developmental dysplasia of the hip. The principal finding was that THA after PAO differed most markedly from primary THA, showing higher cup placement and a greater need for bulk bone grafts and reinforcement rings, whereas the shelf and Chiari procedures produced more modest deviations.

Although cup inclination, anteversion, and CE angle were comparable across cohorts, the PAO group exhibited a significantly more proximal hip center (mean cup height 29 mm vs. 22 mm in controls). An elevated hip center after PAO has been reported [26], and is generally attributed to the anterolateral reorientation of the acetabular fragment and relative superior migration of the true acetabular floor during osteotomy healing [21,22]. In our series, the Chiari group also trended toward a higher hip center (27.8 mm), reflecting medial displacement of the acetabular roof that reduces superior coverage and obliges the surgeon to raise the cup to restore offset, which is essential for maintaining joint stability and abductor muscle tension [27,28]. Because a proximitized hip center increases joint-reaction force and may accelerate polyethylene wear, surgeons should consider larger-diameter heads or dual-mobility constructs to mitigate instability and edge loading in such cases. Elevated hip centers have been associated with increased rates of polyethylene wear, reduced implant survivorship, and a higher risk of revision due to biomechanical disadvantages and instability [29]. For instance, Bicanic et al. found that elevated hip centers correlated with increased polyethylene wear and aseptic loosening in long-term follow-up of dysplastic hips treated with THA, underscoring the importance of careful cup height selection during preoperative planning [24,30,31]. Proper acetabular cup positioning is critical in THA, as it directly influences joint stability, range of motion, and long-term implant survival. Malpositioned cups may lead to complications such as dislocation, impingement, limb length discrepancy, and accelerated wear. Although this study did not directly assess postoperative dislocation rates, previous reports suggest that a proximally and laterally displaced hip center, as commonly seen after PAO, may increase joint-reaction forces and reduce soft tissue tension, potentially raising the risk of instability. In such cases, the use of larger femoral heads or dual-mobility constructs may help reduce the risk of dislocation and improve mid- to long-term implant survival [24,32,33,34]. Therefore, understanding how prior pelvic osteotomy affects cup orientation and placement is essential for optimizing outcomes in this challenging subset of patients [25,27].

Operative time was longest in the PAO cohort and showed a clinically relevant, albeit non-significant, increase of ~30 min relative to controls [35]. While the difference did not reach statistical significance in our current sample, the 30 min increase in operative time may become significant with larger cohorts. Prolonged operative duration is a known risk factor for perioperative complications, including surgical site infection, and may also impact anesthesia duration, surgeon fatigue, and operating room scheduling efficiency [36]. Thus, even modest increases in surgical time warrant consideration in preoperative planning for post-osteotomy THA. The higher frequency of bulk femoral-head autografts and support rings in the PAO group confirms that residual segmental defects are common after Bernese PAO [37], consistent with meta-analytic data indicating graft utilization rates of 10–25% [21]. Although blood loss did not differ significantly, the combination of prolonged surgery and additional reconstructive steps underscores the need for meticulous preoperative planning, including three-dimensional computed tomography templating and availability of modular augments.

Although we initially hypothesized that THA following Chiari osteotomy would result in greater technical challenges due to medial displacement of the acetabular roof and extensive soft tissue scarring, our findings revealed that the PAO group exhibited more substantial deviations in cup positioning and a higher frequency of reconstructive device use. This discrepancy may be explained by the anterolateral reorientation of the acetabulum after Bernese PAO, which often leads to superior bone defects and altered landmarks that complicate component placement. These structural changes likely increase the technical demands of THA in post-PAO cases more than previously appreciated.

Taken together, our results suggest that the type of index osteotomy should inform both implant selection and alignment targets. After PAO, elevation of the hip center may be unavoidable; therefore, attention should shift to restoring global offset and ensuring stable fixation with adjunctive hardware when necessary [38,39]. In Chiari cases, medialization of the acetabular component may compromise combined anteversion, potentially increasing the risk of posterior impingement and instability [40,41,42]. In contrast, shelf procedures largely preserve the native acetabular architecture, resulting in radiographic and surgical parameters comparable to those of primary THA [43,44].

To reduce the risk of acetabular malposition in patients with prior pelvic osteotomies, several technical strategies should be considered. Preoperative three-dimensional templating using CT imaging can help evaluate acetabular morphology and anticipate segmental defects [45]. Intraoperatively, accurate identification of the true acetabular floor is essential, particularly when native landmarks are distorted by prior osteotomy or grafting [46]. In such cases, intraoperative fluoroscopy or computer-assisted navigation may improve cup alignment [47]. Additionally, using modular acetabular systems and offset reamers allows greater flexibility in managing complex anatomy [21]. Incorporating these techniques may help improve cup placement accuracy and optimize clinical outcomes in this challenging subset of patients.

Strengths of this work include direct, head-to-head comparison of three osteotomy subtypes, use of a contemporaneous primary-THA control, and acceptable measurement reliability (ICC = 0.91). This study has several limitations. First, its retrospective design is subject to inherent biases, including potential selection and information bias. Second, the surgical procedures were performed over an extended period, during which advances in implant design, surgical techniques, and perioperative care may have influenced the outcomes. Although the use of tranexamic acid has been reported to be effective for hemostasis [48], its administration was not evaluated in the present study. Third, the operations were conducted by different surgeons, introducing variability in surgical approach and decision-making. A total of six experienced orthopedic surgeons performed the procedures during the study period. Although surgical technique varied somewhat between individuals, all cases were conducted under a standardized institutional protocol that guided acetabular cup placement based on anatomical landmarks and preoperative imaging, which helped reduce inter-surgeon variability. Fourth, the sample size was small—particularly in the Chiari group—which may limit the statistical power and generalizability of the results. Although postoperative complications such as dislocation were reviewed, the small sample size and low event rate precluded meaningful statistical comparison across groups. Larger studies are needed to clarify whether altered cup position after osteotomy affects complication rates. Fifth, although computed tomography may provide more accurate measurements than radiography [49], we used radiographs for postoperative assessment due to concerns about radiation exposure. Moreover, the utility of radiographic measurements has been supported in a previous study [50]. Furthermore, standard anteroposterior pelvic radiographs are widely used in clinical practice and have been shown to provide reliable measurements of cup inclination and vertical height from the inter-teardrop line. Several studies have demonstrated that these parameters correlate with surgical accuracy and patient outcomes, and that radiographic evaluations offer high interobserver agreement, making them suitable for comparative analyses in retrospective cohorts. Finally, clinical outcome measures such as validated functional scores (e.g., Harris Hip Score) were not available, preventing direct correlation between radiographic findings and patient-reported outcomes.

Prospective, multicenter studies with larger cohorts are warranted to confirm these findings and define thresholds for clinically significant hip-center elevation. Integration of computer-assisted navigation or patient-specific instrumentation may further refine component placement in the anatomically distorted acetabulum. Finally, long-term follow-up is needed to clarify whether the higher hip center seen after PAO translates into increased wear, instability, or aseptic loosening.

## 5. Conclusions

Cup position and intra-operative requirements in THA vary according to the type of prior pelvic osteotomy. PAO is associated with proximal cup placement and a higher likelihood of bone-grafting and reinforcement devices, highlighting the technical demands of this subgroup. Surgeons should tailor surgical strategy and implant selection to the specific anatomic alterations created by each osteotomy to optimize THA outcomes. For example, after PAO, surgeons may consider the use of reinforcement rings or larger-diameter femoral heads to address superior segmental defects and mitigate instability due to a higher hip center. In Chiari cases, careful management of combined anteversion and consideration of medialized cup placement are important to avoid posterior impingement. In contrast, shelf procedures often allow for standard press-fit cup placement due to preservation of the native acetabular architecture.

## Figures and Tables

**Table 1:** Patient demographics and baseline characteristics.

Parameter	PAO Group (n = 12)	Chiari Group (n = 5)	Shelf Group (n = 8)	Control Group (n = 25)	*p*-Value (vs Control)
Sex (Male/Female)	1/11	1/4	3/5	4/21	0.52/0.82/0.19
BMI (kg/m^2^)	24.1 ± 3.0	23.7 ± 1.4	23.0 ± 2.5	24.7 ± 4.0	0.91/0.81/0.61
Age at THA conversion (years)	57.3 ± 11.3	63.0 ± 8.7	63.8 ± 8.6	63.6 ± 11.8	0.27/0.99/0.99

**Table 2:** Results of radiographic cup positioning.

Parameter	PAO Group (n = 12)	Chiari Group (n = 5)	Shelf Group (n = 8)	Control Group (n = 25)	*p*-Value (vs Control)
Center-edge angle	24.4 ± 16.3	18.0 ± 12.3	28.2 ± 9.2	18.1 ± 8.4	0.33/0.99/0.21
Inclination angle	45.2 ± 10.4	39.2 ± 5.1	47.2 ± 5.0	43.7 ± 4.9	0.88/0.26/0.63
Anteversion angle	13.9 ± 5.6	9.4 ± 9.5	14.4 ± 10.0	14.7 ± 6.8	0.98/0.16/0.99
Height (mm)	29.0 ± 8.9	27.8 ± 7.5	27.2 ± 3.6	21.8 ± 5.4	0.0075 */0.07/0.21

**Table 3:** Results of surgical parameters.

Parameter	PAO Group (n = 12)	Chiari Group (n = 5)	Shelf Group (n = 8)	Control Group (n = 25)	*p*-Value (vs Control)
Operative time (min)	123.0 ± 58.0	97.2 ± 33.9	107.2 ± 27.1	93.1 ± 24.6	0.078/0.99/0.82
Blood loss (cc)	499.0 ± 472.4	329.2 ± 173.6	549.3 ± 295.1	466.0 ± 311.7	0.94/0.68/0.99
Bulk bone graft use	2 (16.7%)	1 (12.5%)	0 (0%)	0 (0%)	0.036 */0.07/1.0
Support ring use	2 (16.7%)	0 (0%)	0 (0%)	0 (0%)	0.036 */1.0/1.0

## Data Availability

The data that support the findings of this study are available upon request from the corresponding author.

## Abbreviations

The following abbreviations are used in this manuscript:

THA	Total hip arthroplasty
DDH	Developmental dysplasia of hip
PAO	Periacetabular osteotomy
CE	Center-edge
ICC	Intraclass Correlation Coefficient
AP	Anteroposterior

## References

[1] Jacobsen S., Sonne-Holm S. (2005). Hip dysplasia: A significant risk factor for the development of hip osteoarthritis. A cross-sectional survey. Rheumatology.

[2] Reijman M., Hazes J.M.W., Pols H.A.P., Koes B.W., Bierma-Zeinstra S.M.A. (2005). Acetabular Dysplasia Predicts Incident Osteoarthritis of the Hip The Rotterdam Study. Arthritis Rheum..

[3] Sato T., Yamate S., Utsunomiya T., Inaba Y., Ike H., Kinoshita K., Doi K., Kawano T., Shiomoto K., Hara T. (2024). Life Course Epidemiology of Hip Osteoarthritis in Japan A Multicenter, Cross-Sectional Study. J. Bone Jt. Surg. Am..

[4] Southam L., Rodriguez-Lopez J., Wilkins J.M., Pombo-Suarez M., Snelling S., Gomez-Reino J.J., Chapman K., Gonzalez A., Loughlin J. (2007). An SNP in the 5′-UTR of *GDF5* is associated with osteoarthritis susceptibility in Europeans and with in vivo differences in allelic expression in articular cartilage. Hum. Mol. Genet..

[5] Masuya H., Nishida K., Furuichi T., Toki H., Nishimura G., Kawabata H., Yokoyama H., Yoshida A., Tominaga S., Nagano J. (2007). A novel dominant-negative mutation in *Gdf5* generated by ENU mutagenesis impairs joint formation and causes osteoarthritis in mice. Hum. Mol. Genet..

[6] Mototani H., Mabuchi A., Saito S., Fujioka M., Iida A., Takatori Y., Kotani A., Kubo T., Nakamura K., Sekine A. (2005). A functional single nucleotide polymorphism in the core promoter region of *CALM1* is associated with hip osteoarthritis in Japanese. Hum. Mol. Genet..

[7] Nakajima M., Kizawa H., Saitoh M., Kou I., Miyazono K., Ikegawa S. (2007). Mechanisms for asporin function and regulation in articular cartilage. J. Biol. Chem..

[8] Feldman G.J., Parvizi J., Sawan H., Erickson J.A., Peters C.L. (2014). Linkage Mapping and Whole Exome Sequencing Identify a Shared Variant in *CXCR3* in a Large Multi-Generation Family. J. Arthroplast..

[9] Mori Y., Ueno K., Chiba D., Hashimoto K., Kawai Y., Baba K., Tanaka H., Aki T., Ogasawara M., Shibasaki N. (2023). Genome-Wide Association Study and Transcriptome of Japanese Patients with Developmental Dysplasia of the Hip Demonstrates an Association with the Ferroptosis Signaling Pathway. Int. J. Mol. Sci..

[10] Dygut J., Piwowar M. (2025). Distinction Between Dysplasia, Malformation, and Deformity-Towards the Proper Diagnosis and Treatment of Hip Development Disorders. Diagnostics.

[11] Wen J., Ping H., Kong X., Chai W. (2023). Developmental dysplasia of the hip: A systematic review of susceptibility genes and epigenetics. Gene.

[12] Bakarman K., Alsiddiky A.M., Zamzam M., Alzain K.O., Alhuzaimi F.S., Rafiq Z. (2023). Developmental Dysplasia of the Hip (DDH): Etiology, Diagnosis, and Management. Cureus.

[13] Venkatadass K., Durga Prasad V., Al Ahmadi N.M.M., Rajasekaran S. (2022). Pelvic osteotomies in hip dysplasia: Why, when and how?. EFORT Open Rev..

[14] Ganz R., Klaue K., Vinh T.S., Mast J.W. (1988). A new periacetabular osteotomy for the treatment of hip dysplasias. Technique and preliminary results. Clin. Orthop. Relat. Res..

[15] Cirrincione P., Cao N., Trotzky Z., Nichols E., Sink E. (2024). Does Periacetabular Osteotomy Change Sagittal Spinopelvic Alignment?. Clin. Orthop. Relat. Res..

[16] Bartonicek J., Vavra J., Chochola A. (2012). Bosworth hip shelf arthroplasty in adult dysplastic hips: Ten to twenty three year results. Int. Orthop..

[17] Tanaka H., Chiba D., Mori Y., Kuwahara Y., Baba K., Yamada N., Fujii G., Itoi E. (2018). Long-term results of a modified Spitzy shelf operation for developmental dysplasia of the hip in adults and adolescents. Eur. J. Orthop. Surg. Traumatol..

[18] Chiari K. (1953). Pelvic osteotomy in hip arthroplasty. Wien. Med. Wochenschr..

[19] Kurishima H., Chiba D., Baba K., Hamada S., Suzuki T., Kanabuchi R., Fujii G., Oyama M., Ochiai T., Mori Y. (2024). Long-term results of Chiari pelvic osteotomy on the preservation of hip function with mean follow-up of more than 30 years and its prognostic factors. J. Orthop. Sci..

[20] Jawad M.U., Scully S.P. (2011). In brief: Crowe’s classification: Arthroplasty in developmental dysplasia of the hip. Clin. Orthop. Relat. Res..

[21] Huan S.W., Wu W.R., Peng S.J., Zhuang T.F., Liu N. (2024). Total hip arthroplasty after pelvic osteotomy: A meta-analysis. Acta Orthop. Belg..

[22] Shapira J., Annin S., Rosinsky P.J., Maldonado D.R., Lall A.C., Domb B.G. (2021). Total hip arthroplasty after pelvic osteotomy for acetabular dysplasia: A systematic review. J. Orthop..

[23] Henderson E.R., Marulanda G.A., Cheong D., Temple H.T., Letson G.D. (2011). Hip abductor moment arm--a mathematical analysis for proximal femoral replacement. J. Orthop. Surg. Res..

[24] Göktaş H., Subaşi E., Uzkut M., Kara M., Biçici H., Shirazi H., Chethan K.N., Mihçin Ş. (2022). Optimization of Hip Implant Designs Based on Its Mechanical Behaviour. Biomechanics in Medicine, Sport and Biology.

[25] Lewinnek G.E., Lewis J.L., Tarr R., Compere C.L., Zimmerman J.R. (1978). Dislocations after total hip-replacement arthroplasties. J. Bone Jt. Surg. Am..

[26] Fukui K., Kaneuji A., Sugimori T., Ichiseki T., Matsumoto T. (2015). Does rotational acetabular osteotomy affect subsequent total hip arthroplasty?. Arch. Orthop. Trauma. Surg..

[27] Sarin V.K., Pratt W.R., Bradley G.W. (2005). Accurate femur repositioning is critical during intraoperative total hip arthroplasty length and offset assessment. J. Arthroplast..

[28] Tanaka H., Yamada N., Kurishima H., Mori Y., Aizawa T. (2022). Association between Hip Center Position and Isokinetic Hip Muscle Performance after Anterolateral Muscle-Sparing Total Hip Arthroplasty. Medicina.

[29] Wu C., Shu G., Xie X., Yuan X., Chen S. (2022). Meta-analysis of the Efficacy of the Anatomical Center and High Hip Center Techniques in the Treatment of Adult Developmental Dysplasia of the Hip. Biomed. Res. Int..

[30] Bicanic G., Delimar D., Delimar M., Pecina M. (2009). Influence of the acetabular cup position on hip load during arthroplasty in hip dysplasia. Int. Orthop..

[31] Hidayat T., Ammarullah M.I., Saputra E., Lamura M.D.P., K N C., Ismail R., Bayuseno A.P., Jamari J. (2024). A method for estimating the contact area of a dual-mobility total hip prosthesis. AIP Adv..

[32] Cho M.R., Choi W.K., Kim J.J. (2016). Current Concepts of Using Large Femoral Heads in Total Hip Arthroplasty. Hip Pelvis.

[33] Kostensalo I., Junnila M., Virolainen P., Remes V., Matilainen M., Vahlberg T., Pulkkinen P., Eskelinen A., Makela K.T. (2013). Effect of femoral head size on risk of revision for dislocation after total hip arthroplasty: A population-based analysis of 42,379 primary procedures from the Finnish Arthroplasty Register. Acta Orthop..

[34] Houcke J.V., Khanduja V., Pattyn C., Audenaert E. (2017). The History of Biomechanics in Total Hip Arthroplasty. Indian. J. Orthop..

[35] Komiyama K., Hamai S., Motomura G., Ikemura S., Fujii M., Kawahara S., Nakashima Y. (2021). Total hip arthroplasty after periacetabular osteotomy versus primary total hip arthroplasty: A propensity-matched cohort study. Arch. Orthop. Trauma. Surg..

[36] Sikov M., Sloan M., Sheth N.P. (2021). Effect of operative time on complications following primary total hip arthroplasty: Analysis of the NSQIP database. Hip Int..

[37] Tamaki T., Oinuma K., Miura Y., Shiratsuchi H. (2016). Total Hip Arthroplasty after Previous Acetabular Osteotomy: Comparison of Three Types of Acetabular Osteotomy. J. Arthroplast..

[38] Parvizi J., Burmeister H., Ganz R. (2004). Previous Bernese periacetabular osteotomy does not compromise the results of total hip arthroplasty. Clin. Orthop. Relat. Res..

[39] Yuasa T., Maezawa K., Nozawa M., Kaneko K. (2015). Total hip arthroplasty after previous rotational acetabular osteotomy. Eur. J. Orthop. Surg. Traumatol..

[40] Tokunaga K., Aslam N., Zdero R., Schemitsch E.H., Waddell J.P. (2011). Effect of prior Salter or Chiari osteotomy on THA with developmental hip dysplasia. Clin. Orthop. Relat. Res..

[41] Chiari C., Schneider E., Stamm T., Peloschek P., Kotz R., Windhager R. (2024). Ultra-long-term results of the Chiari pelvic osteotomy in hip dysplasia patients: A minimum of thirty-five years follow-up. Int. Orthop..

[42] Minoda Y., Kadowaki T., Kim M. (2006). Total hip arthroplasty of dysplastic hip after previous Chiari pelvic osteotomy. Arch. Orthop. Trauma. Surg..

[43] Ikezaki T., Kawai T., Okuzu Y., Goto K., Kuroda Y., Matsuda S. (2024). Effects of prior shelf procedure on subsequent conversion total hip arthroplasty. BMC Musculoskelet. Disord..

[44] Willemsen K., Doelman C.J., Sam A.S.Y., Seevinck P.R., Sakkers R.J.B., Weinans H., van Der Wal B.C.H. (2020). Long-term outcomes of the hip shelf arthroplasty in adolescents and adults with residual hip dysplasia: A systematic review. Acta Orthop..

[45] Bishi H., Smith J.B.V., Asopa V., Field R.E., Wang C., Sochart D.H. (2022). Comparison of the accuracy of 2D and 3D templating methods for planning primary total hip replacement: A systematic review and meta-analysis. EFORT Open Rev..

[46] Shigemura T., Yamamoto Y., Murata Y., Sato T., Tsuchiya R., Wada Y. (2018). Total hip arthroplasty after a previous pelvic osteotomy: A systematic review and meta-analysis. Orthop. Traumatol. Surg. Res..

[47] Romanelli F., Hong I.S., Khan J.A., Porter A., Jankowski J.M., Liporace F.A., Yoon R.S. (2024). Intraoperative Fluoroscopy Versus Navigation to Determine Cup Anteversion in Direct Anterior Total Hip Replacement: A Technical Trick for Obtaining “True” Anteversion. Arthroplast. Today.

[48] Tanaka H., Tarasawa K., Mori Y., Baba K., Kanabuchi R., Kuriyama Y., Kurishima H., Fukuchi H., Kawamata H., Fushimi K. (2025). Tranexamic acid in total hip arthroplasty: Nationwide evidence for reducing blood transfusions and post-operative complications. J. Jt. Surg. Res..

[49] Ghelman B., Kepler C.K., Lyman S., Della Valle A.G. (2009). CT outperforms radiography for determination of acetabular cup version after THA. Clin. Orthop. Relat. Res..

[50] Mori Y., Mori N., Mori T., Nakamura S., Ishizuka M., Sano T., Itoi E. (2014). Risk of acetabular protrusion is low in rheumatoid arthritis patients treated with bipolar hemiarthroplasty for displaced femoral neck fractures without rheumatoid change in hip joints. Eur. J. Orthop. Surg. Traumatol..

